# Biobank-based genetic characterization of neurodegenerative diseases and idiopathic normal pressure hydrocephalus: insights and lessons learned from FinnGen

**DOI:** 10.1038/s41380-026-03676-3

**Published:** 2026-06-12

**Authors:** Sami Heikkinen, Valtteri Julkunen, Henna Martiskainen, Mari Takalo, Eino Solje, Ville Leinonen, Annakaisa Haapasalo, Anssi Lipponen, Mikko Hiltunen

**Affiliations:** 1 Institute of Biomedicine, University of Eastern Finland, Kuopio, Finland; 2 Institute of Clinical Medicine, University of Eastern Finland, Kuopio, Finland; 3 Neuro Center, Kuopio University Hospital, Kuopio, Finland; 4 A.I. Virtanen Institute for Molecular Sciences, University of Eastern Finland, Kuopio, Finland

**Keywords:** Neuroscience, Genetics

## Abstract

Brain disorders characterized by progressive neurodegeneration, such as Alzheimer’s disease (AD) and frontotemporal dementia (FTD), represent an increasing medical and societal challenge. While genome‑wide studies have uncovered numerous susceptibility loci, these efforts have largely focused on common variants and leave a substantial portion of genetic liability unresolved. Variants of low frequency, often associated with stronger biological effects, remain insufficiently characterized, particularly in heterogeneous populations. Genetically isolated populations offer an effective strategy to overcome these limitations. Finland, shaped by historical demographic events, harbors a distinctive spectrum of enriched rare variants that can facilitate gene discovery. The FinnGen initiative capitalizes on this setting by combining extensive genotyping with nationwide health registry data through a coordinated network of Finnish biobanks. With half a million participants analyzed, FinnGen supports highly powered analyses across a broad array of clinical outcomes and registry data. Recent comprehensive analyses have reported thousands of significant genotype–phenotype associations, including novel protein‑altering variants. Importantly, the FinnGen cohort structure favors older individuals and hospital‑derived samples, increasing representation of brain disorders, such as AD and idiopathic normal pressure hydrocephalus (iNPH), a disorder frequently accompanied by AD‑like pathological features. In this expert review, we summarize FinnGen‑based investigations relevant to neurodegenerative diseases and iNPH, highlighting insights into genetic susceptibility, disease overlap, and protective factors, and discuss how integration with recall studies as well as biomarker and clinical data accelerates translational applications in brain disorders.

## Introduction

Neurodegenerative diseases, including Alzheimer’s disease (AD), frontotemporal dementia (FTD), and related disorders, represent a growing global health challenge due to their progressive nature, high prevalence, and lack of curative treatments [1–3]. After decades of research, the genetic architecture underlying these conditions is beginning to be resolved. Large-scale genome-wide association studies (GWAS) have identified numerous common variants associated with neurodegenerative diseases, yet many of these explain only a fraction of heritability. Rare and low-frequency variants, which often exert larger effects, are particularly difficult to capture in multi-ethnic cohorts. This gap underscores the need for population-based resources that can illuminate the full spectrum of genetic risk. Finland offers a unique opportunity for such research. As a population isolate shaped by historical bottlenecks and founder effects in Finland [4–6], the Finnish gene pool is enriched for rare and low-frequency variants that are virtually absent elsewhere. These variants can provide critical insights into underlying disease mechanisms, therapeutic targets, and biomarker discovery.

Leveraging this genetic landscape, the FinnGen project was launched as a nationwide initiative to integrate genomic data with comprehensive health registry information in Finland (https://www.finngen.fi/en). FinnGen is built on a nationwide network of Finnish biobanks, which form the backbone of the project. Finland currently has 11 registered biobanks, including hospital‑based biobanks, population‑based biobanks, and the Finnish Red Cross Blood Service Biobank. These biobanks operate under a common legal and ethical framework defined by the Finnish Biobank Act and are supervised by the Finnish Medicines Agency. With ~500,000 individuals analyzed to date, FinnGen combines biobank samples with longitudinal clinical data, enabling robust association studies across a wide range of phenotypes. The recent landmark analysis from FinnGen exemplifies the power of this approach [4]. By examining more than 1900 disease endpoints, the study identified thousands of genome-wide significant associations, including numerous novel loci and coding variants with large effect sizes. These findings highlight the value of population isolates for uncovering genetic determinants of complex diseases. Importantly, the design in FinnGen enriches older individuals and hospital-based recruitment, increasing the prevalence of neurodegenerative diseases and other brain disorders, such as idiopathic normal pressure hydrocephalus (iNPH). Individual-level data collected by FinnGen is available for custom analysis by registered users of FinnGen partners. FinnGen-generated GWAS summary statistics for about 2800 predefined endpoints are publicly available after an embargo of one year (https://risteys.finngen.fi).

Together, these features position FinnGen as a uniquely powerful platform for elucidating the genetic basis of neurodegenerative diseases and other brain disorders. The enrichment of rare and low‑frequency variants in the Finnish population, combined with deep phenotyping and long‑term registry follow‑up, enables systematic identification of both shared and distinct genetic risk factors. Here, we summarize FinnGen‑related research activities conducted so far with a particular focus on neurodegenerative diseases and iNPH. We discuss how FinnGen has been leveraged to investigate disease risk, resilience, and overlap across these conditions, and outline future opportunities for integrating genetic discoveries with biomarkers and clinical data to advance translational applications in brain disorders.

## The evolving architecture of AD genetics in FinnGen

### Meta-analysis of AD genetics

The landscape of AD genetics has undergone a period of rapid refinement driven by ever-larger genome-wide association studies (GWAS) and increasingly sophisticated analytical methods [7–13]. Among recent efforts that have included also FinnGen data are the European-focused meta-analyses by Bellenguez et al. [14] and the AD Consensus Meta-analysis [15], which together form the backbone of our contemporary understanding of common-variant AD risk. These studies are complemented by earlier large-scale work, such as Wightman et al. [16] and by more recent, and much needed, ancestry-diverse analysis exemplified by Lake et al. [17]. A common thread across these studies is the growing influence of the FinnGen resource, whose deeply phenotyped Finnish cohort repeatedly enhances statistical power, refines locus resolution, and anchors cross-study harmonization.

The study by Bellenguez et al. represents a transformational advance in AD genetics [14]. Leveraging the extensive European Alzheimer & Dementia Biobank (EADB) and incorporating UK Biobank proxy-AD data alongside a large replication stage including FinnGen, ADGC, and CHARGE, this study assembled one of the largest ever European-ancestry AD datasets. The resulting discovery of seventy-five genome-wide significant loci, of which forty-two were previously unreported, marked a decisive expansion of the known genetic architecture of AD. FinnGen’s contribution was particularly important here: many of the emerging loci achieved genome-wide significance only because FinnGen provided sufficient replication evidence, often in genomic regions where allele frequencies or haplotype structures differ slightly from other European cohorts.

Importantly, the use of a multilayered gene prioritization framework reinforced the implication of microglial biology, and more specifically amyloid processing, tau pathology, lysosomal and endocytic pathways, and immune-cell biology in AD. Altogether, Bellenguez et al. provided a comprehensive and biologically interpretable atlas of AD risk loci, setting a new standard for both discovery and functional interpretation [14].

Beyond reaffirming *TREM2* and the *MS4A* locus as major microglial risk components, Bellenguez et al. highlighted *e.g. PLCG2*, which encodes phospholipase Cγ2, a key intracellular signaling effector downstream of TREM2 and other microglial ITAM-coupled receptors. Notably, the rare missense variant *PLCG2* p.P522R (rs72824905 C > G) confers protection against AD and other neurodegenerative diseases, consistent with functional studies showing that the P522R substitution produces a mildly hypermorphic enzyme that enhances microglial phagocytic responses and resilience to inflammatory stress [18, 19]. The effects of AD-associated *PLCG2* and *TREM2* variants on AD risk and onset age have been examined in the FinnGen cohort [20]. The *PLCG2* p.P522R and a newly identified *PLCG2*-3’ UTR variant (rs4243226 A > G) associated with the decreased AD risk and delayed onset, including among heterozygous *APOE4* carriers. In contrast, the *TREM2* p.R62H AD risk variant associated with earlier onset age including among *APOE4* carriers. While *PLCG2* p.P522R shows modest effect sizes in pan‑European meta‑analyses, its protective effect is substantially stronger in FinnGen (OR = 0.53, 95% CI 0.37–0.75) as compared to Bellenquez *et al*. [14] (OR = 0.74, 95% CI 0.68–0.81). This highlights a broader observation that microglial signaling genes often show population-specific effect-size variation, and that FinnGen’s haplotypic homogeneity can sharpen genetic signals that otherwise remain diffuse. Similarly, in a recent preprint Koivumäki et al. replicated the AD-association of *ABI3* p.S209F risk variant using FinnGen data and demonstrated that the risk allele associated with earlier AD onset [21]. This study further employed recall of FinnGen participants to investigate the effects of *ABI3* p.S209F on neuroimaging endophenotypes.

Although *TYROBP* has not yet been implicated through common-variant meta-analyses, its centrality to microglial immunity makes it an important gene in the broader AD genetics landscape. *TYROBP* encodes DAP12, the adaptor protein that couples TREM2 and other microglial surface receptors to intracellular ITAM signaling cascades. Disruption of *TYROBP* in mouse models produces defective microglial activation and aberrant amyloid responses, and rare loss-of-function mutations cause Nasu–Hakola disease (NHD), a severe early-onset neurodegenerative disorder characterized by microglial dysfunction [22]. Martiskainen et al. studied a Finnish founder mutation involving a deletion of exons 1–4 in *TYROBP*, which causes NHD, also known as polycystic lipomembranous osteodysplasia with sclerosing leukoencephalopathy (PLOSL) in homozygous carriers [23]. Using FinnGen data, monoallelic *TYROBP* deletion was identified as a novel genetic risk factor for AD and dementia. Moreover, monoallelic *TYROBP* deletion associated with an earlier onset of AD and dementia when compared to noncarriers. Functional studies in cell models indicated that monoallelic *TYROBP* deletion leads to increased inflammatory response, accompanied by downregulation of unfolded protein response and oxidative phosphorylation pathways. The existence of the *TYROBP* deletion illustrates how founder populations can reveal risk or protective variation in canonical microglial signaling axes—variation that remains largely invisible in pan-European datasets with different allele frequency spectra. Together, these studies demonstrate the power of the FinnGen cohort to validate known AD risk variants, discover novel genetic associations, and elucidate the functional consequences of the variants.

The Consensus Meta-analysis harmonizes the results of Bellenguez et al., Wightman et al. and additional datasets into a unified framework [15]. This effort addressed a critical need within the field: the major AD GWAS had been conducted with different phenotype definitions, QC procedures, and analytical choices, which resulted in minor but important discrepancies in reported loci and effect sizes. By integrating these studies under a uniform analytical umbrella, the Consensus meta-analysis produced a stable reference dataset for *e.g*. polygenic risk modeling, fine-mapping, functional genomics, and cross-ancestry comparison. FinnGen again enhanced the stability of this harmonized dataset. Its consistent case-control definitions, large sample size, and distinct but well-characterized LD structure substantially improved the precision of effect-size estimates. The Consensus project thus can be seen as a culmination—and consolidation—of the European-based AD association landscape, providing a mature reference against which emerging studies can be benchmarked. These achievements are best understood in light of Wightman et al. [16]. This study was the first to scale AD GWAS to more than a million individuals by combining clinically diagnosed cases and parental proxy cases from several cohorts, including an early FinnGen data release. It identified thirty-eight genome-wide significant loci, including seven that had not been reported previously, and provided influential early evidence for the centrality of microglial function and immune signaling in late-onset AD. However, the Wightman et al. study lacked the extensive replication, functional mapping, and standardized processing that characterize the later Bellenguez and Consensus studies. Indeed, many of the loci identified by Wightman are now incorporated into the Consensus dataset, but their effect-size estimates are more stable due to 30% higher case or proxy case count. FinnGen’s presence in Wightman et al. was helpful, but its role became substantially more consequential in the later studies, where its increasingly larger number of AD cases and controls repeatedly anchor replication and fine-mapping efforts.

A parallel development is represented by the multi-ancestry meta-analysis from Lake et al. [17], which responds to the long-standing need to move beyond the European-centric view of AD genetics. This study integrates AD GWAS summary statistics from European, African American, East Asian, and Caribbean Hispanic populations and applies ancestry-aware statistical methods. The results highlight both shared and ancestry-specific components of AD risk. Two loci not identified in the European-dominated studies that include FinnGen—near *TRANK1* and *VWA5B2*—suggest that the global genetic architecture of AD contains elements not detectable in European-only samples. FinnGen contributes to this study by strengthening the European-based anchor against which ancestry-dependent deviations in effect size can be interpreted.

### Chromosome X GWAS (XWAS) in AD

Two recent large-scale studies have independently addressed the long-standing exclusion of the X chromosome from Alzheimer’s disease (AD) genetics, demonstrating that X-linked variation contributes detectable, albeit modest, effects to AD risk. In parallel efforts, Le Borgne et al. and Belloy et al. each performed X-chromosome–wide association studies (XWASs) using extensive, partly overlapping collections of clinically diagnosed and registry-based AD cohorts [24, 25]. Both studies showed that, despite substantial statistical and technical challenges, X-linked loci can be reliably interrogated at biobank scale and yield biologically interpretable signals.

Le Borgne et al. identified several loci reaching X-chromosome–wide significance and emphasized that a subset of X-linked variants may contribute to sex-dependent risk architectures, consistent with broader evidence for sex differences in AD prevalence and progression. Independently, Belloy et al. reported the X-linked AD association at *SLC9A7*, supported by colocalization with brain and immune expression quantitative trait loci, implicating endosomal and Golgi pH regulation as a novel pathway in AD pathogenesis. Notably, though, despite some overlap in study cohorts, including FinnGen, Le Borgne et al. did not report this locus. Both studies also provided evidence that some X-linked loci escape X-chromosome inactivation, reinforcing the biological plausibility of X-chromosome involvement in AD. In both analyses, FinnGen again contributed by improving statistical power, strengthening cross-cohort consistency, and demonstrating that well-phenotyped national biobanks can meaningfully advance also X-chromosome genetics.

### New perspectives on APOE-driven AD risk

Two recent studies refine our understanding of *APOE*’s role in AD, together underscoring the centrality of apolipoprotein E to disease burden. In the first study, Williams et al. quantify the population-level impact of *APOE* by estimating the proportion of AD and all-cause dementia attributable to *APOE* genotypes (population attributable fraction, PAF) across multiple large datasets, including UK Biobank, FinnGen, the A4 Study, and ADGC autopsy-confirmed cases [26]. Using *APOE2* homozygotes (*APOE22)* as the low-risk reference group, the authors demonstrate that the combined effects of *APOE3* and *APOE4* account for the majority of AD cases, with estimates ranging from approximately two-thirds of clinically diagnosed AD in FinnGen to over 90% of neuropathologically confirmed AD in ADGC. A particularly provocative conclusion is that *APOE3*, often treated as a neutral reference allele, contributes substantially to AD burden at the population level when compared to the low-risk reference group of *APOE22*. FinnGen plays a key role in this analysis by providing a large, well-ascertained national cohort with long follow-up and registry-based diagnoses, yielding PAF estimates that are both precise and broadly consistent across the cohort.

In the second study, Thomassen et al. provide, through a large *APOE*-stratified, cross-ancestry GWAS meta-analysis in AD, a detailed view into the genetics that modifies disease susceptibility beyond the canonical *APOE2/3/4* framework [27]. This work found no loci associated with AD in the *APOE22* + *32* stratum, contrasting the ten loci associated with AD in both *APOE33* and *APOE44* + *43* strata, all of which are known AD loci, like *BIN1*, *MS4A64*, or *TREM2*. Importantly, this work also identified strata-specific loci, nine in *APOE44* + *43* (5 known, 4 novel) and nine in *APOE33* (6 known, 3 novel). Pathway analysis of the GWAS loci suggests that the genetic risk of AD beyond *APOE* may relate to immunity especially in the *APOE4* carriers. This study highlights mechanisms through which *APOE* biology may be modulated in different populations and clinical contexts. FinnGen again contributes importantly here by offering enriched haplotypes and rare variants that are difficult to study in more heterogeneous European cohorts. Together, these studies reinforce *APOE*’s dominant role in AD while demonstrating that both population-level burden and fine-grained genetic architecture must be considered to fully understand—and ultimately target— *APOE*-driven disease risk.

### Polygenic risk of AD

As genome-wide association studies have progressively expanded the catalog of Alzheimer’s disease (AD) risk loci, polygenic risk scores (PRS)—also referred to as genetic risk scores or polygenic hazard scores—have emerged as a key framework for translating diffuse genetic signals into individual-level risk estimates. Early PRS applications in AD demonstrated that aggregating common variants beyond APOE captures meaningful additional heritable risk, but recent studies have substantially refined both their construction and interpretation.

Large GWAS meta-analyses now provide the backbone for contemporary AD PRS. In the European Alzheimer & Dementia Biobank (EADB) study by Bellenguez et al., PRS derived from genome-wide significant and suggestive loci showed clear stratification of future AD risk and progression from mild cognitive impairment, even after accounting for *APOE4* status [14]. Importantly, individuals in the highest PRS deciles exhibited approximately 1.6–1.9-fold increased risk relative to those in the lowest deciles, underscoring that polygenic burden meaningfully modifies susceptibility on top of age and *APOE*. FinnGen again contributed independent validation and improved calibration of effect sizes.

The recent consensus meta-analysis of AD and related dementias extended this framework by constructing PRS that explicitly excluded *APOE* and were based on rigorously defined loci [15]. These scores were shown to associate primarily with neuropathological hallmarks of AD—Braak neurofibrillary tangle stage and neuritic β-amyloid plaque burden—rather than with non-AD dementias or cerebrovascular pathology. Notably, individuals in the top PRS decile had roughly two-fold increased odds of severe AD neuropathology at autopsy compared with those in the median group. This work clarified that non-APOE polygenic burden reflects core AD biology and is not merely a proxy for diagnostic heterogeneity introduced by large biobank or proxy-case designs.

Methodological refinements to PRS construction are addressed in several complementary studies that include FinnGen data. Sofer et al. demonstrated that PRS performance depends strongly on variant inclusion thresholds and phenotype definitions, with liability-scale variance explained remaining modest but reproducible across cohorts [28]. Nicolas et al. further showed that incorporating rare variants and pathway-informed weighting can modestly enhance predictive performance, particularly in younger individuals or those lacking *APOE4* [29]. Akdeniz et al. and Okorie et al. explored PRS transferability across populations and clinical contexts, emphasizing that ancestry mismatch and cohort ascertainment can substantially attenuate predictive accuracy [30, 31].

The convergence of these findings suggests that PRS are best viewed not as standalone diagnostic tools, but as scalable instruments for risk stratification, enrichment of prevention trials, and dissection of AD heterogeneity when integrated with age, *APOE*, biomarkers, and clinical trajectories.

When viewed collectively (Table 1), the above-described AD GWAS and PRS meta-analyses in FinnGen illustrate the progression of AD genetics from initial large-scale association efforts, through comprehensive European-based mapping and functional interpretation, to the beginnings of a global, ancestry-inclusive framework. The Bellenguez and Consensus studies emerge as the pivotal reference works in this trajectory, providing both the scale and the methodological sophistication necessary to stabilize and contextualize discoveries from earlier efforts and to support the expansion into multi-ancestry territory. Throughout this evolution, FinnGen has become an increasingly important resource. Across studies, it consistently increases statistical power, improves locus resolution through its distinct haplotype structure, enhances replication fidelity, and supports the harmonization of diverse datasets into a coherent portrait of AD genetic architecture. As the field continues to expand toward more comprehensive multi-ancestry representation and deeper mechanistic integration, FinnGen is poised to remain central to high-quality, large-scale discovery.

**Table 1:** Summary of the introduced studies on the genetics of AD including FinnGen data.

Section	Reference	Study focus	Key findings	FinnGen-% (cases / controls)^a^	Role of FinnGen
Meta-analysis of AD genetics	Bellenguez et al. [14]	Large European GWAS meta-analysis	Identified 75 AD loci (42 novel); strong microglial and immune pathway enrichment	5.6 / 36.2	Provided a large, biobank-based European cohort that substantially increased statistical power for locus discovery and replication, improved fine-mapping through Finnish-specific LD structure, and served as a stabilizing anchor for cross-study harmonization in large GWAS meta-analyses.
	ADGC et al. [15]	Harmonized AD GWAS meta-analysis	Produced stable reference set of AD loci and effect sizes for downstream analyses	6.0 / 39.4	
	Wightman et al. [16]	Earlier large-scale AD GWAS	Identified 38 loci; highlighted immune and microglial biology	2.0 / 7.0	
	Lake et al. [17]	Multi-ancestry AD meta-analysis	Identified ancestry-specific loci (TRANK1, VWA5B2); modeled effect heterogeneity	7.3 / 24.1	
Microglial rare and founder variants	Jeskanen et al. [20]	*PLCG2* and *TREM2* variants	*PLCG2* protective variants delay AD onset; *TREM2* risk variant accelerates onset	100 / 100	Enabled the detection and characterization of rare, population-enriched, and founder variants in key microglial genes, allowing stronger effect-size estimation, age-at-onset analyses, APOE-stratified effects, and functional follow-up that are difficult to achieve in heterogeneous European cohorts.
	Koivumäki et al. [21]	ABI3 p.S209F variant		100 / 100	
	Martiskainen et al. [23]	TYROBP monoallelic deletion	Identified monoallelic *TYROBP* deletion as AD risk factor with earlier onset	100 / 100	
Chromosome X GWAS (XWAS)	Le Borgne et al. [24]	X-chromosome GWAS in AD	Identified X-linked loci; suggested sex-dependent risk architecture	6.5 / 29.3	Contributed large numbers of well-phenotyped AD cases from national registries, increasing power and consistency for analytically challenging X-chromosome analyses and supported the identification and validation of sex-linked AD risk loci.
	Belloy et al. [25]	X-chromosome GWAS in AD	Identified SLC9A7 as X-linked AD locus; implicated endosomal pH regulation	11.3 / 39.1	
Beyond APOE ε4	Williams et al. [26]	APOE population attributable fraction	Showed APOE ε3 and ε4 explain majority of AD burden	62.4 / 62.1	Supported both population-level and fine-grained APOE analyses by providing long follow-up, reliable registry-based diagnoses, enriched haplotypes, and rare variants that sharpen the estimation of APOE-attributable risk and modifier effects beyond ε2/ε3/ε4.
	Thomassen et al. [27]	APOE-stratified GWAS	Identified ε4- and ε3-specific AD loci; immunity pathways prominent in ε4 carriers	29.4 / 49.0	
Polygenic risk of AD^b^	Bellenguez et al. [14]	PRS from AD GWAS	PRS stratifies AD risk beyond APOE; top deciles ~1.6–1.9× risk	5.6 / 36.2	Contributed to the large GWAS meta-analyses from which the PRS were derived, enabling the validation of polygenic risk and hazard scores and the assessment of robustness and portability in independent cohorts.
	ADGC et al. [15]	APOE-excluded PRS	Non-APOE PRS associates with AD neuropathology	6.0 / 39.4	
	Sofer et al. [28]	PRS methodology	PRS performance sensitive to variant thresholds and phenotype	3.3 / 24.9	
	Nicolas et al. [29]	PRS transferability	European PRS less transferable across ancestries	5.6 / 36.2	
	Akdeniz et al. [30]	Polygenic hazard score	PHS predicts age-specific AD risk	12.7 / 45.8	

Within each table section, the studies are listed in the order of appearance within the text.

^a^Out of the total study sample sizes. Of note, the total size of FinnGen and the number of AD cases has increased towards the newer studies.

^b^In all PRS studies FinnGen contributed GWAS summary statistics for the PRS derivation.

## Recall studies in FinnGen: rationale, implementation, and lessons learned

Key challenges in medical studies and drug development are the efficient screening and recruitment of suitable participants. In AD clinical trials, traditional methods are often slow, costly, and inefficient, with screening-failure rates of up to 78–88% [32]. While FinnGen provides a uniquely large and well-characterized genomic and registry-based cohort it lacks certain types of clinical data streams such as large-scale proteomic and routine biomarker collections needed for deep phenotyping and many hypothesis-driven translational studies. Recall studies —where biobank participants are recontacted for additional phenotyping — address these limitations by enabling targeted, fast collection of new data tied to rich longitudinal registry information.

The first recall initiatives in 2022 piloted the feasibility of recontacting FinnGen participants and examined factors influencing participation and the use of easily applicable screening methods for determining cognitive status and biomarker discovery [33, 34]. In these studies, the final participation rates were about 19% of all subjects contacted with elderly individuals responding less frequently than the young. However, in [33] the enrollment rates were originally substantially higher (~39%), but the COVID 19 pandemic reduced final completion rates in the frail study population. Invitation method strongly affected response: mailed letters outperformed digital portals, and reminders increased response rates. Although absolute participation was modest, data quality was high, and remote cognitive assessments combined with blood-based AD biomarkers showed promise as cost effective alternatives to traditional in person evaluations [33].

Motivated by these pilots, a larger recall based on FinnGen and the Finnish Twins Cohort – the TWINGEN study – was launched in 2023 [35]. TWINGEN is a nationwide Finnish biobank recall study designed to identify non-demented older adults at increased risk of AD by integrating blood-based biomarkers, cognitive assessments (both remote and in-person), and lifestyle data from up to 50 years of prior follow-up. By leveraging the biobank infrastructure, TWINGEN demonstrated the feasibility of large-scale recall for deep phenotyping and AD risk stratification. In TWINGEN the median recall-rate was higher (~30% and reaching ~50% in some areas in Finland) compared to the earlier pilot studies. Recruitment was rapid — distribution of ~3000 invitation letters yielded ~700 participants completing the protocol across multiple sites within months — illustrating the speed and reach possible with a nationwide biobank network [35]. Initial TWINGEN analyses reported that plasma phosphorylated tau 217 (p-tau217) is highly heritable (≈56%) and strongly associated with age and *APOE4* status, supporting its potential as a scalable marker of AD pathology in individuals without dementia [36]. A TWINGEN based validation study also found that a telephone administered multitrial word list learning task reliably measures episodic memory, converges with in person testing, and discriminates between cognitively healthy and impaired participants [37].

**Fig. 1:**
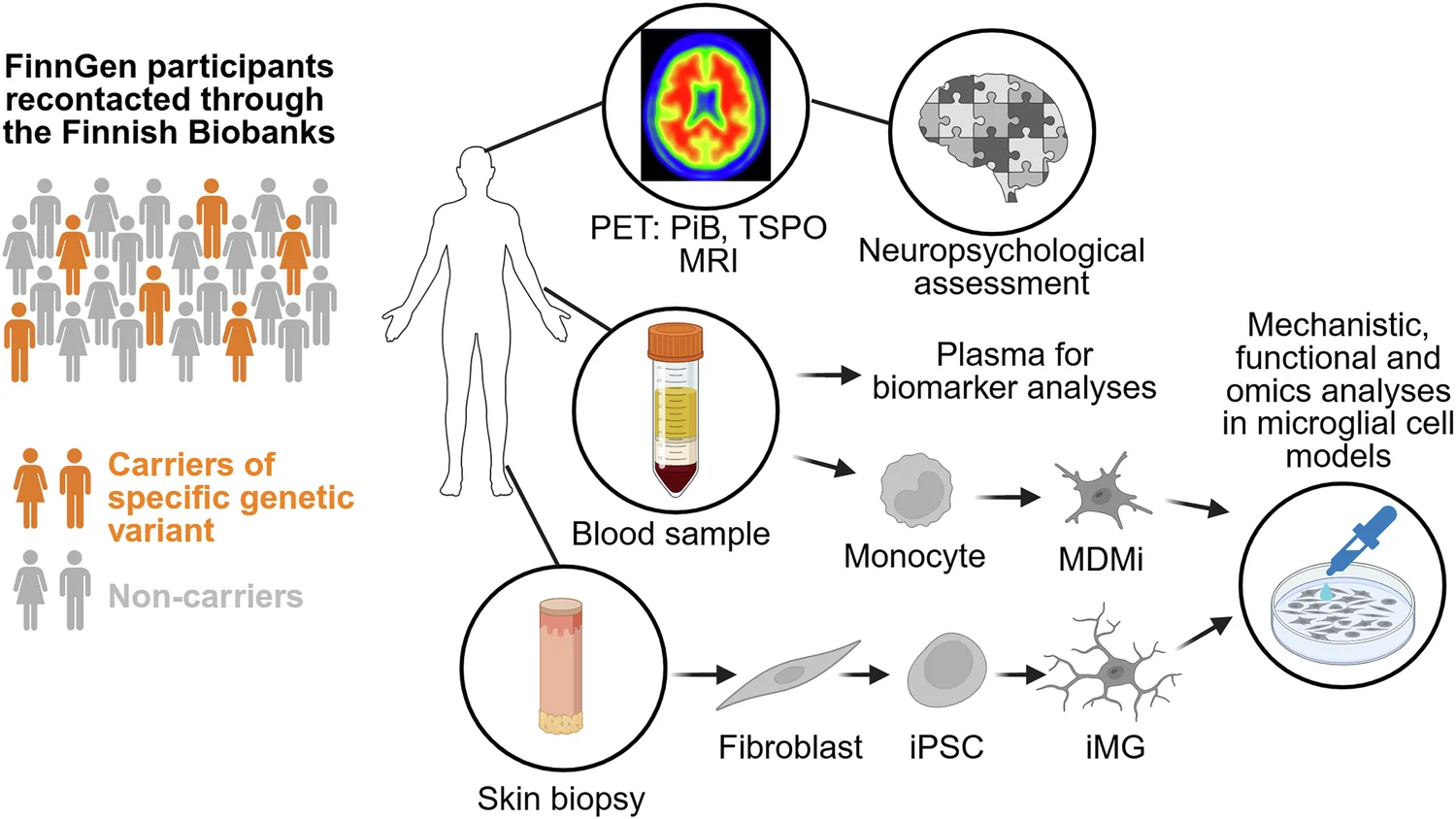
Schematic overview of the recall study strategy for investigating specific genetic variants. FinnGen genotype data can be used to identify eligible variant carriers and non-carriers, who are recontacted through the Finnish biobanks. Participants who consent and meet the study-specific inclusion criteria are invited to a recall visit that can include PET and MRI imaging, neuropsychological assessment, and collection of blood and skin biopsies. Blood is processed to obtain plasma for biomarker analyses and monocytes for generating monocyte-derived microglia-like cells (MDMi). Fibroblasts from skin biopsies are reprogrammed into induced pluripotent stem cells (iPSC) and further differentiated into induced microglia (iMG). These cellular models enable mechanistic, functional, and omics analyses. Created in BioRender, https://BioRender.com/2x58aie.

Subsequent recall studies have extended the approach to carriers of specific genetic risk variants. For example, recalls targeting *ABI3*, *PLCG2*, and *TYROBP* variant carriers enabled detailed mechanistic, clinical and biomarker characterization of individuals at elevated risk for neurodegenerative disease (Fig. 1) [21, 23, 38]. Because these variants are exceptionally rare (minor allele frequencies in FinnGen ~0.002–0.008), nationwide biobank resources are essential for identifying carriers and enabling translational studies that would otherwise be infeasible. In the *ABI3* study, cognitively healthy carriers of p.S209F AD risk variant (aged ≥ 50 years, *n* = 58) were recalled based on genotype information. These participants underwent comprehensive clinical and biomarker assessments, including cognitive testing, brain MRI, and amyloid and microglia PET imaging [21]. During the recall visits, skin biopsies were collected for fibroblast isolation and iPSC generation, alongside blood sampling for plasma biomarker analysis and monocyte isolation. Both iPSCs and blood-derived monocytes were subsequently used to produce microglia-like cell models, enabling functional studies of variant-specific mechanisms (manuscript in preparation). A similar approach was applied to characterize the *PLCG2* p.P522R variant, which confers protection against neurodegenerative diseases and cognitive decline [38]. In the *TYROBP* study, carriers of a large structural variant (a > 5 kb deletion) could not be directly identified from the available FinnGen genotype data. Therefore, existing DNA samples from biobanks were used to validate proxy markers before recalling, ensuring accurate detection of deletion carriers [23]. The proxy markers demonstrated high specificity for the deletion, and their imputed genotypes were reliable when high-quality imputation data were available. During the subsequent recall, samples for iPSC generation and blood-derived monocyte isolation were collected [23]. This illustrates a dual strategy of leveraging existing biobank samples and conducting targeted recalls only when necessary.

Furthermore, data collected in recall projects can be reintegrated into FinnGen database, which is updated regularly. This enables longitudinal follow-up of recall cohorts without repeated recontact, facilitating predictive modeling and outcome tracking at lower cost. Together, these examples illustrate the possibilities of FinnGen and the Finnish biobanks as a platform for biomarker discovery and targeted subject selection paving the way for cost-effective early detection and recruitment strategies in dementia research and biobank-based recall studies.

Recall studies carry practical and regulatory challenges. Based on our experience, planning and regulatory approvals commonly take 1–2 years from concept to study initiation, whereas active data collection can often be completed within a few months. Recall rates are variable and can be low, especially among frail older adults and those without access to digital portals. Invitation modality matters: physical letters plus reminders consistently outperform single mode digital invitations. Lightweight remote screening methods (telephone cognitive tests, remote questionnaires, blood-based biomarkers) can increase efficiency and reduce costs, but careful validation against in person standards is essential. Despite these obstacles, nationwide recalls using biobank infrastructure show substantial potential to accelerate clinical and translational research. Recall studies can bridge clinical research, registry-based epidemiology, and basic science by providing targeted deep phenotyping linked to long term health records, thereby supporting precision medicine approaches and more efficient trial recruitment strategies.

## FinnGen applications focusing on NPH/iNPH

### NPH/iNPH genetics

The genetics of NPH and iNPH have gradually moved from isolated candidate gene findings toward population-scale understanding. Earlier studies were based on small, selected NPH/iNPH cohorts and typically used approaches such as whole exome or whole genome sequencing, familial case analyses, copy number variation studies, or Mendelian randomization [39, 40]. These studies identified loss-of-function variants in *CWH43*, *CFAP43*, *DNAH14*, structural changes in SFMBT1 and, through whole genome sequencing, also in *AK9, RXFP2, PRKD1, HAVCR1, OTOG, MYO7A, NOTCH1, SPG11*, and *MYH13* [41]. The FinnGen analyses provide a contrasting perspective by assessing risk variants in a nationwide dataset enabling systematic testing of both common and low frequency variants.

### NPH/iNPH cohort definition

The health registry data available in FinnGen allowed careful stratification of idiopathic and secondary NPH. Patients with the NPH were identified by ICD10 code G91.2, and those with prior diagnoses indicating potential secondary causes such as intracranial hemorrhage, tumors, trauma, stroke, infection, congenital malformations or postprocedural etiologies were excluded to align with the American European iNPH guidelines and the Relkin criteria to identify the idiopathic NPH patients [42, 43]. Although this approach improves specificity, it may exclude some true iNPH cases. Controls consisted of FinnGen participants without hydrocephalus diagnoses.

### NPH genetics findings

The FinnGen NPH GWAS initially identified eight genome-wide significant loci [44]. Although the replication using UK Biobank data was limited by the small number of confirmed NPH cases, six of the eight loci nevertheless showed consistent effects in the meta-analysis (NPH n: FinnGen = 1522, UKBB = 173). The top loci were *SLCO1A2* (lead variant rs7962263), *AMZ1/GNA12* (rs798495), *MLLT10* (rs10828247), *CDCA2* (rs561699566 and rs371919113), *C16orf95* (rs56023709) and *PLEKHG1* (rs62434144).

*SLCO1A2* encodes a solute carrier involved in organic anion transport. Its proximity to a protective variant suggests that transport across fluid regulating barriers, such as the blood–brain barrier or blood–CSF barrier, could influence CSF homeostasis [45, 46]. Expression analyses show that the protective allele increases *SLCO1A2* expression in cerebellar tissue, further supporting its potential functional role [44]. *AMZ1/GNA12* genes play roles in intracellular signaling [47]. *AMZ1* is expressed in several brain regions while *GNA12* is expressed mainly in peripheral tissues [44]. *GNA12* encodes a G-protein alpha subunit involved in RhoA signaling, a pathway previously linked to ventricular size regulation and vascular development in the periventricular germinal matrix [47, 48]. These functional associations suggest mechanisms that link *AMZ1/GNA12* variation to CSF dynamics [44]. *MLLT10* represents another protective locus. The effect allele of rs10828247 increases expression of *CASC10* in the cerebellum and colocalizes with non-brain eQTLs for both *MLLT10* and *NEBL* [44]. The locus contains several regulatory elements and multiple candidate genes, indicating a potential influence on transcriptional regulation relevant to brain tissue. The *CDCA2* locus includes two intronic variants (rs561699566 and rs371919113), and although the locus has not been functionally characterized in NPH, the finemapping credible sets suggest possible regulatory effects. *C16orf95* has been associated previously with increased lateral ventricular volume, aligning well with NPH/iNPH pathophysiology in which ventriculomegaly is a defining feature [49]. *PLEKHG1* represents a risk locus with unique colocalization patterns. Its association appears specific to NPH among neurological or systemic traits, suggesting a potentially distinct biological contribution, possibly linked to cytoskeletal or barrier related pathways [44]. Surprisingly, the GWAS did not identify significant associations at any of the previously proposed *CWH43* [50], *SFMBT* [51], *CFAP43* [52] or *DNAH14* [53] loci, indicating that earlier findings may not generalize to broader populations.

In the dedicated FinnGen study of *CWH43* loss-of-function variants, carrier frequencies in FinnGen were 3.1% among iNPH patients and 2.5% among controls, with no significant effect on disease risk or onset age. Furthermore, none of the carriers had a hydrocephalus diagnosis [54]. Similarly low variant frequencies and non-association to clinical phenotype or shunt responsiveness replicated in a Norwegian cohort [54]. These findings collectively suggest that the previously reported *CWH43* associations from small cohorts do not represent major risk determinants at the population level at least in Finland.

### Use of individual genotypes and follow-up data of NPH patients

Individual-level FinnGen genotype data has already enabled us to evaluate whether putative risk variants including *CWH43* loss-of-function alleles affected age-at-onset of NPH [54]. In the future, individual-level data allows stratification of cases by comorbidities, CSF and plasma biomarker levels, proteomics, imaging, drug purchases and clinical trajectories, as well as allele-dose modelling. These analyses illustrate how FinnGen’s longitudinal national registry linkage and high-quality genotyping can be leveraged to investigate both rare and common variants contributing to iNPH. Since FinnGen is periodically updated with the Finnish population-based registries, the new diagnoses further sharpen the genetic findings.

The health-care registries available in FinnGen allow for careful determination of the specific diagnosis which is warranted since different etiologies are expected in different hydrocephalus subtypes [55]. For example, the FinnGen-based GWAS-study suggests that in late-onset hydrocephalus, et al., NPH, the findings are rather related to brain fluid clearance and ventricle volume than cilia abnormalities seen in earlier onset hydrocephalus. Somewhat unexpectedly, however, similar odds ratios were found in NPH, the iNPH subset and the further shunted iNPH subset, suggesting that a similar genetic risk profile underlies also secondary NPH and therefore also the susceptibility to it after any severe CNS insult. Furthermore, FinnGen may help identify thus far unknown environmental factors that potentially lead to the development of hydrocephalus in subjects with susceptibility-increasing genetic profiles.

## Genetic studies of FTD and ALS in FinnGen

FTD and amyotrophic lateral sclerosis (ALS) are neurodegenerative diseases forming a disease spectrum characterized by overlapping genetic background, clinical symptoms, and neuropathology [56]. The onset age in the FTD-ALS spectrum diseases typically varies between 40–70 years, however, FTD is the second most common cause of early-onset dementia in individuals younger than 65 years of age [57]. The *C9orf72* hexanucleotide repeat expansions (*C9-HRE*) are the most common Mendelian mutations causing FTD and ALS in Northern Europe [58–60], and are particularly prevalent in Finnish FTD and ALS patients. Previous studies have indicated that approximately 46% of familial ALS and 48% familial FTD cases in Finland are caused by *C9-HRE* [58, 61]. FinnGen has not been extensively utilized in FTD and ALS research, but there are a few reports on FinnGen-based studies in these disorders.

FinnGen has been utilized to identify a SNP or a haplotype that could be used as a genetic proxy marker for identifying potential *C9-HRE* carriers in large population-based genetic cohorts [62]. First, a genotype-confirmed cohort of C9-HRE carriers in the EADB database was used to identify *C9-HRE*-associated SNPs and haplotypes. The SNP rs139185008 alone or haplotypes containing this SNP was found to strongly associate with both intermediate-length (15–30 repeats) and long (>60 repeats) *C9-HRE* carriers. rs139185008 was then tested in FinnGen (data release 5, containing data from 218,792 individuals), where it significantly associated with FTD and ALS clinical endpoints in Finns. rs139185008 was in complete linkage disequilibrium with a previously reported *C9-HRE*-associated surrogate marker, rs3849942, and significantly associated with ALS in UK Biobank, validating the findings. However, in UK Biobank, it was not among the top associating SNPs, highlighting population-specific differences in the haplotype structures of the associated SNPs. These data suggest that this genetic proxy marker can help identify potential *C9-HRE* carriers without labor-intensive testing in large population-based cohorts, such as GWAS, where complex genetic alterations, such as large repeat expansions or deletions cannot directly be detected. The carrier status of *C9-HRE* in the identified individuals can then be further confirmed by targeted genotyping.

In another study, Kaivola et al. used a Finnish case-control cohort of ALS cases and controls to identify the best SNPs for tagging the intermediate-length *C9-HRE* [63]. They previously showed using this same cohort that carriership of two copies of the intermediate-length alleles, especially when one of the alleles is > 17 repeats in length, confers increased risk for ALS [64]. They described that rs2814707*T (minor allele) was the best tagging SNP for intermediate-length *C9-HRE* alleles with ≥ 7 repeats and the previously identified rs139185008*C [62] for the long C9-HRE as well as alleles with ≥ 20 repeats [63]. They then used these SNPs in FinnGen (data release 10) to examine the effect of the intermediate-length alleles on longevity and found that rs139185008*C frequency decreased with age. The decrease started after 70 years of age when ALS and FTD diagnoses were excluded, suggesting that the rs139185008*C haplotype may also increase the risk of other late-onset neurodegenerative diseases as well [63]. Beyond C9-HRE proxy marker approaches, several studies have leveraged FinnGen in Mendelian Randomization (MR) frameworks to pinpoint causal relationships increasing or decreasing the risk for ALS or FTD.

Kong et al. investigated if type 2 diabetes mellitus (T2DM) affects the risk of ALS [65]. By using MR, they discovered that T2DM exhibited a protective effect on ALS. By analyzing blood metabolites, they found that the protective effect involved lipid but not glucose pathways, suggesting especially total esterified cholesterol and low-density lipoprotein particle diameter as mediators of the protective effect. These findings propose that targeting lipid metabolism might offer new therapeutic avenues to manage ALS risk.

Cheng et al. utilized MR to study causal relationship between immune cells and FTD [66]. They detected that 21 immune phenotypes have a causal relationship with FTD pathogenesis; 13 of them being protective and eight associated with increased risk. The protective immune phenotypes included various T cell and B cell markers, including those of Tregs, previously indicated e.g., in anti-inflammatory functions. The risk-increasing phenotypes were related to markers of myeloid cells and subsets of T and B cells linked to functions such as promotion of neuroinflammation. Conversely, FTD did not exert a significant causal effect on immunophenotypes. The results provide insights into possible roles of specific immune cell types in FTD pathogenesis and are in line with several previous studies indicating immune system alterations in different subtypes of FTD.

Altered circulating hormones in ALS have been reported in some previous studies. Fan et al. conducted an MR study to evaluate whether the hormonal factors insulin-like growth factor-1 (IGF-1); sex hormone-binding globulin (SHBG); free testosterone (FT); total testosterone (TT); and estradiol affect the risk of ALS [67]. In addition, they screened 90 circulating proteins, such as cytokines, chemokines, growth factors, and interferons, using existing data from European cohorts to evaluate their risk effect on ALS. FinnGen summary data of ALS were used in the replication analyses in the study. Higher level of FT was found to associate with a 23% reduced risk of ALS. Screening of proteomic traits indicated 12 plasma proteins causally associated with ALS. Genetic predisposition to higher levels of plasma IL27, IL-1ra, Dkk-1, PSGL-1, MMP7, PECAM-1, and ST2 associated with lower risk of ALS, while HSP-27, CA-125, cathepsin D, MMP1, and U-PAR associated with increased ALS risk. These findings may provide novel biomarker and therapeutic target candidates and underline potential mechanistic pathways in ALS.

Finally, a recent preprint investigated using MR the causal relationship between thyroid function traits (TSH, FT4, autoimmune hyperthyroidism, autoimmune hypothyroidism) and ALS [68]. A causal effect of autoimmune hyperthyroidism on reduced ALS risk was detected, suggesting that genetic predisposition to autoimmune hyperthyroidism may decrease risk of ALS. No converse causal effects of ALS on thyroid hormones or hypo- or hyperthyroidism were found.

Together, these studies demonstrate that FinnGen is a powerful resource for further studies related to the genetic background and risk factors as well as causal co-morbidities affecting disease pathogenesis of ALS and FTD. As FinnGen continues to grow, its unique founder population genetics and comprehensive clinical data will increasingly enable high-resolution mapping of disease mechanisms and precision medicine development in both FTD and ALS.

## Conclusions and future prospectives

By combining the unique population genetic structure of Finland with nationwide longitudinal health data, FinnGen enables the identification of robust, biologically grounded risk signals that can be translated into disease mechanisms, biomarkers, and therapeutic targets. However, the use of large population‑based biobank cohorts in the genetic meta‑analyses highlights important methodological considerations. Biobank datasets often have less precise clinical phenotype definitions and highly imbalanced case–control ratios, with large excesses of controls. While this substantially increases statistical power, it may also disproportionately influence effect estimates in meta-analyses and introduce population‑specific biases, if not carefully accounted. Thus, inclusion of more than one biobank, explicit reporting of cohort‑level case–control balance, along with sensitivity and stratified analyses, should be considered as best practices when integrating population‑based cohorts into large genetic consortia. The critical next steps for FinnGen relate to the systematic integration of genetic findings with disease‑relevant endophenotypes, including biofluid and imaging biomarkers, medication use patterns, and other longitudinal clinical markers, which provide direct insight into underlying pathophysiology, disease stage, and treatment response. Such integrative approaches enhance causal inference, refine risk stratification, and allow genetic associations to be interpreted in a clinically meaningful context. Importantly, these combined data enable the identification of asymptomatic individuals at high risk for neurodegenerative diseases and other brain disorders well before clinical onset, creating unprecedented opportunities for early intervention. In the future prospective, it would be advantageous to enable efficient recruitment of individuals identified through FinnGen into genotype‑ and endophenotype‑guided observational studies and clinical trials evaluating disease‑modifying strategies. This approach would facilitate a transition from late‑stage symptomatic treatment toward early, mechanism‑based interventions.
